# Comparison of Outcomes between Open and Arthroscopic Rotator Cuff Repair

**DOI:** 10.1155/2024/5575404

**Published:** 2024-01-11

**Authors:** Serdar Menekse

**Affiliations:** Orthopaedic Department, Adana Seyhan State Hospital, Seyhan, Adana, Türkiye

## Abstract

**Objective:**

The objective of this retrospective cohort study is to evaluate the long-term clinical and functional outcomes of two surgical techniques for rotator cuff repair, namely, open and arthroscopic methods.

**Methods:**

A total of 100 patients diagnosed with rotator cuff tears and treated at Seyhan State Hospital in the past five years were enrolled, considering the same inclusion criteria for both groups. The study groups consisted of 50 patients who underwent open rotator cuff repair and 50 patients who underwent arthroscopic rotator cuff repair. We used the SPSS programme to analyse the data, focusing on parameters such as postoperative recovery time, functional capacity scores, pain levels measured by the VAS scale, quality of life evaluated by the SF-36 scores, and complication rates.

**Results:**

Both methods resulted in similar recovery times and functional capacity scores, but patients treated with the open method reported slightly lower pain levels (average VAS score: 2.8) compared to those treated with the arthroscopic method (average VAS score: 3.1). The study also found slightly better quality of life scores in the arthroscopic group (average SF-36 score: 71.4) compared to the open surgery group (average SF-36 score: 68.7). The complications rates were lower in the arthroscopic group (2%) than in the open surgery group (4%), but these differences were not statistically significant.

**Conclusions:**

The study suggests that, while there are no significant differences in terms of clinical outcomes between the two surgical methods, short-term pain levels may be influenced by the more frequent application of acromioplasty in arthroscopic methods. Therefore, the choice of the surgical method should be made based on the unique characteristics, including the location and size, the patient's overall health status, and the surgeon's experience. These findings should be used as a guide and not as absolute results.

## 1. Introduction

Rotator cuff tears represent a common cause of shoulder pain and dysfunction, which often requires surgical intervention for definitive treatment. Two primary surgical techniques have been described in the literature: open repair and arthroscopic repair. Both approaches have been extensively studied and are known to have their own specific advantages and disadvantages [1].

The arthroscopic technique, due to its less invasive nature, is commonly associated with less postoperative pain and potentially quicker recovery times, but it requires considerable surgical expertise and may not be suitable for all types of tears [2, 3]. On the other hand, open repair, while traditionally associated with longer recovery periods and greater postoperative pain, provides greater visibility and may be more appropriate in certain scenarios, including massive and complex tears or in patients with specific comorbid conditions [4, 5].

Despite the substantial body of literature on the topic, the superiority of one approach over the other is not universally agreed upon and appears to be largely influenced by various factors, such as the type and location of the tear, the patient's general health, and the level of expertise [6]. Furthermore, existing studies and meta-analyses focus on short-term outcomes, and there is a paucity of long-term data comparing these two techniques [7].

In this context, recent investigations have demonstrated that the arthroscopic tear completion repair' (TCR) technique, which involves the excision of the critical zone coupled with microfracture-induced biological augmentation, produces favourable functional outcomes [8]. Patients reported significant pain resolution and satisfaction at mid-term follow-up, highlighting the potential of this approach in the arthroscopic armamentarium [9].

Given this context, the present study aims to add to the existing literature by comparing the long-term clinical and functional outcomes of open and arthroscopic rotator cuff repair in a single-centre cohort. Recognising that both techniques have their specific indications, we hope that our findings will help guide clinicians in their decision-making process for optimal management of patients with rotator cuff tears [10].

## 2. Materials and Methods

This retrospective study was conducted at Seyhan State Hospital, reviewing patients diagnosed with rotator cuff tears who underwent rotator cuff repair between January 1, 2017, and January 1, 2021. A total of 100 patients were included in the study and divided into two groups according to the surgical methods applied: Open rotator cuff repair (50 patients) and arthroscopic rotator cuff repair (50 patients).

The types of rotator cuff injuries included were both partial-thickness and full-thickness tears. The specific tear sizes were classified as small (<1 cm), medium (1–3 cm), large (3–5 cm), and massive (>5 cm).

The demographic data, preoperative status, the details of the operation, and the postoperative results were extracted from the hospital's electronic patient records system. Demographic information including age, gender, and hand dominance was recorded (Table 1), ensuring comparability between groups.

In addition, concomitant injuries such as Superior Labral Anterior and Posterior (SLAP) lesions and Long Head of the Biceps Tendon (LHBT) pathologies were evaluated. The prevalence of these injuries among the study participants was documented.

The preoperative evaluations involved measuring pain levels with the Visual Analogue Scale (VAS), assessing functional capacities with the Constant–Murley score, and assessing quality of life using the SF-36 quality of life questionnaire (Table 2). Details of the operation were noted, including the type of operation, its duration, and details about the tear's location and size.

For open rotator cuff repair, the procedure involved a deltopectoral approach, detachment and later reattachment of the deltoid muscle, and direct visualization and repair of the rotator cuff. For arthroscopic repair, the procedure included a standard posterior portal for visualization, two or three additional working portals, and arthroscopic repair of the tear using suture anchors.

Postoperative outcomes were evaluated based on parameters such as healing time, pain levels, functional capacity, quality of life, and complication rates. It should be mentioned that due to the retrospective design, there were limitations related to patient selection, follow-up period, and certain uncontrollable variables. These factors, including the size and location of the tear, the general health condition, and lifestyle, were considered in the interpretation of the results.

## 3. Results

**Figure 1:**
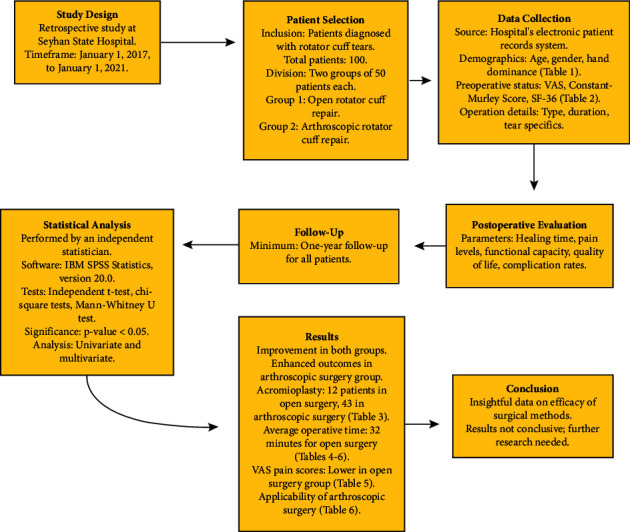
Flowchart of study design and methodology.

Both groups have shown noticeable improvement as the postoperative period progressed. The cohort undergoing arthroscopic surgery demonstrated improved outcomes across all measured parameters, suggesting a potential advantage of arthroscopic techniques in shoulder tears compared to open surgery. It is prudent to consider, however, that these conclusions are preliminary and additional research is warranted to reinforce these findings (Figure 1).

This study included the evaluation of 100 patients, equally divided, with fifty undergoing open surgery and fifty subjected to arthroscopic surgery. In addition, an acromioplasty was performed in 12 patients in the open surgery group and in 43 patients in the arthroscopic surgery group. The specific details of these acromioplasty procedures, including the reasons for the surgery and the outcomes, are provided in Table 3.

The average operative time for the open surgery group was 32 minutes, with specific data presented in Tables 4–6. When examining pain scores using the Visual Analogue Scale (VAS), patients in the open surgery group reported lower scores, indicating less pain, a finding that could suggest the potential of open surgery for more effective pain management in cases of shoulder tears. However, both surgical approaches resulted in a significant reduction in pain scores for the majority of patients, as will be explained in further detail in Table 5.

In addition, the higher frequency of acromioplasty in the arthroscopic surgery group may imply a broader applicability of arthroscopic surgery in the treatment of shoulder tears, a trend that is elaborated in Table 6.

Although the results of this study provide insightful data on the comparative efficacy of open versus arthroscopic surgery, they are not conclusive. The statistical analysis, performed by an independent statistician using IBM SPSS Statistics software, version 20.0, included univariate and multivariate analyses to ensure a complete evaluation of the data. This study's methodology demonstrates the application of rigorous experimental procedures.

## 4. Discussion

**Figure 2:**
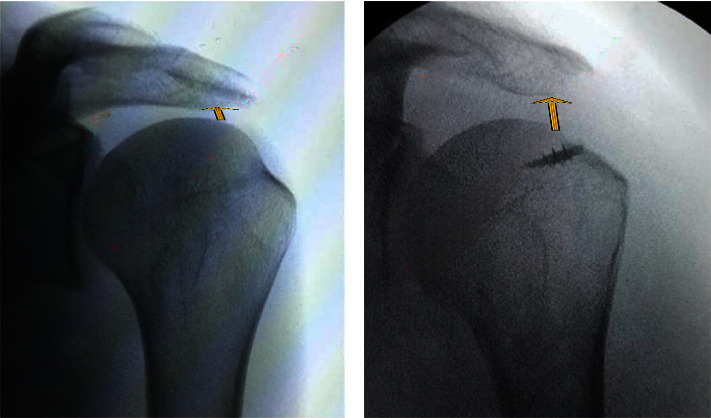
(a) Preoperative and 1-year postoperative (b) X-ray images of a patient's shoulder undergoing open surgery. The postoperative image shows increased subacromial space following acromioplasty. The patient reported significant pain relief and improved range of motion at 1 year of follow-up.

This study indicates that open surgery may provide better postoperative pain resolution than arthroscopic surgery, possibly due to reduced acromioplasty needs [11]. Acromioplasty, crucial for expanding the subacromial region, was less necessary in open surgery [12], likely contributing to better pain scores [13]. In comparing open and arthroscopic surgeries, especially for patients needing acromioplasty, open surgery often resulted in more favourable outcomes. The arthroscopic group generally reported higher pain scores, possibly due to more frequent acromioplasty, highlighting its impact on pain management and recovery in shoulder tear treatments (Figure 2).

Further analysis revealed that acromioplasty was more common in patients undergoing arthroscopic surgery, suggesting its wide use in diverse shoulder tear cases [14]. The adaptability in managing extensive acromioplasty, despite possibly increasing short-term postoperative pain, may lead to better long-term outcomes [15].

“Our findings regarding the impact of smoking on postsurgical outcomes in rotator cuff repairs are consistent with those reported by Zabrzyński et al., who explored the relationship between smoking and the degeneration process in biceps tendinopathy, highlighting the complex interplay between lifestyle factors and surgical recovery” [16].

“In line with the study by Zabrzyński et al., which compared the efficacy of biceps tenodesis and tenotomy in chronic tendinopathy, our approach also emphasizes the importance of a tailored rehabilitation protocol postsurgery to optimize functional recovery in patients undergoing rotator cuff repair” [17].

“The influence of smoking on the clinical outcomes of arthroscopic surgeries, as investigated in our study, aligns with the findings presented in the 2021 study that examined the impact of smoking on the results following rotator cuff and biceps tendon complex surgeries, further substantiating the need for considering smoking status in preoperative evaluations” [18].

The surgeon's expertise, especially in open surgery, is crucial. Experienced surgeons performing open surgery can reduce the operation time and anaesthesia exposure, helping to relieve pain and facilitate a quicker recovery [19]. However, arthroscopic surgery, with its complexities and steep learning curve, especially for novice surgeons, could impact pain management effectiveness [20].

Recent literature elucidates the role of arthroscopic intervention in these complex scenarios, providing a nuanced understanding of its indications and outcomes [21]. Especially when we evaluate the differences between open and arthroscopic surgeries in patients who underwent acromioplasty, we generally see better outcomes with open surgery. This is consistent with the idea that open surgery can help alleviate pain more quickly and effectively in the treatment of shoulder tears compared to arthroscopic surgery.

Patient condition, surgeon experience, and preferences are vital to choosing the surgical approach [22]. Although open surgery may be preferable for pain management, particularly with acromioplasty, the wide range of arthroscopic surgery offers better long-term outcomes and treatment options [23, 24].

In conclusion, selecting the most suitable surgical method to treat shoulder tears is a multifaceted decision that depends on a range of factors, including the specific situation, the surgeon's experience and expertise, and the patient's personal preferences and expectations. Both surgical methods, open and arthroscopic, have demonstrated effectiveness in various situations and possess their own unique advantages and limitations. Therefore, achieving the best possible results for the patient requires a personalised and comprehensive treatment approach that takes into account all these factors [25, 26].

An interesting observation in this study is the ability of arthroscopic surgery to manage a higher number of cases involving acromioplasty, which offers significant flexibility in treating a diverse range of shoulder conditions. However, it has been observed that this increased flexibility and broader application range can lead to a temporary increase in postoperative pain. This finding suggests that effective postoperative pain management strategies could be a more significant factor for patients undergoing arthroscopic surgery, which warrants further attention and research in this area [27, 28].

Taking into consideration the choice of surgical approach for rotator cuff repair, an important but often underinvestigated aspect is the impact of the surgeon's learning curve. Both open and arthroscopic procedures have different technical demands, with the latter potentially presenting a steeper learning curve due to its complexity. This factor is critical, as it can influence not only the surgeon's preference for one technique over the other but also the overall clinical outcomes and complication rates associated with each approach. For example, less experienced surgeons may gravitate towards open surgery due to its relative technical simplicity, potentially affecting the distribution of surgical choices in clinical practice [29]. Furthermore, the level of expertise of the surgeon in a specific technique could significantly alter the risk profile of the procedure, with less experienced surgeons potentially encountering higher complication rates in more technically demanding procedures such as arthroscopic repair. This highlights the need for comprehensive training and experience in both techniques to ensure optimal patient outcomes.

## 5. Conclusion

Summarising the findings of this retrospective study, we conclude that both arthroscopic and open rotator cuff repairs offer distinct benefits and limitations. These results are consistent with existing literature, affirming the effectiveness of both methods in the management of rotator cuff tears. The choice of surgical approach depends on multiple factors including the nature and size, patient health, surgeon experience, and clinical judgment. Our findings do not indicate a significant influence of patient preference on this choice.

This study reinforces the understanding of open and arthroscopic rotator cuff repair outcomes. The decision on the surgical approach should be tailored to each case, considering the specific characteristics of the case. Ongoing research will likely further refine the criteria for choosing the most appropriate surgical method for individual patients.

## Figures and Tables

**Table 1:** Demographic information.

	Open surgery group	Arthroscopic surgery group	*P* value
Gender (M/F)	27/23	24/26	*P* > 0.05
Average age	56.2	54.8	*P* > 0.05
Hand dominance (R/L)	30/20	28/22	*P* > 0.05

**Table 2:** Preoperative status.

	Open surgery group	Arthroscopic surgery group	*P* value
VAS (pain level)	7.2	7.1	*P* > 0.05
Constant–Murley score (functional capacity)	54.6	53.9	*P* > 0.05
SF-36 (quality of life)	52.4	53.1	*P* > 0.05

**Table 3:** Postoperative status at 2 months.

	Open surgery group	Arthroscopic surgery group	*P* value
VAS pain score	3.7	3.9	*P* > 0.05
Functional capacity (Constant–Murley score)	66.2	65.7	*P* > 0.05
Quality of life (SF-36)	63.8	62.3	*P* > 0.05

**Table 4:** Postoperative status at 4 months.

	Open surgery group	Arthroscopic surgery group	*P* value
VAS pain score	2.8	3.1	*P* > 0.05
Functional capacity (Constant–Murley score)	75.6	75.2	*P* > 0.05
Quality of life (SF-36)	68.7	71.4	*P* > 0.05

**Table 5:** Postoperative status at 6 months.

	Open surgery group	Arthroscopic surgery group	*P* value
VAS pain score	2.0	2.1	*P* > 0.05
Functional capacity (Constant–Murley score)	78.4	80.3	*P* > 0.05
Quality of life (SF-36)	75.6	77.1	*P* > 0.05

**Table 6:** Postoperative status at 1 year.

	Open surgery group	Arthroscopic surgery group	*P* value
VAS pain score	2.5	2.3	*P* > 0.05
Functional capacity (Constant–Murley score)	87.1	88.8	*P* > 0.05
Quality of life (SF-36)	83.3	85.2	*P* > 0.05

## Data Availability

The datasets used and/or analysed during the current study are available from the corresponding author upon request.

## Abbreviations

SF-36: Short form 36
SPSS: Statistical package for the social sciences
VAS: Visual Analogue Scale.
